# Book Reviews

**Published:** 1816-05

**Authors:** 


### CRITICAL ANALYSIS

### *OF RECENT PUBLICATIONS*

IN THE

### DIFFERENT BRANCHES OF PHYSIC, SURGERY, AND MEDICAL PHILOSOPHY.

---

*Edinburgh Medical and Surgical Journal*, No. XLVI. for April, 1816.

*Medical Topography of New-Orleans; with an Account of the principal Diseases that affected the Fleet and Army on the late Expedition against that City.* Communicated by a Naval Surgeon.

We have read this paper over with peculiar pleasure and satisfaction. The first part, on the topography of the scene of action, is particularly interesting, being in all respects perspicuous, and, we have no reason to doubt, just. At the same time, it should be observed, that we found great occasion to make allowance for a young writer and practitioner, if not a young man; not for any deficiency in correctness, not for any error in practice, but for a little prurience and a little unacquaintedness with some minuter distinctions in diseases. We, however, heartily congratulate the subjects under his care, the service for such an acquisition, and (if he is really young) the profession on the prospect of such an ornament.

In the medical part of the Topography, we meet with nothing but those effects which are to be expected on flat alluvial soils, probably recently formed by the lighter parts of the land through which is the passage of rivers forming, like the " Mississippi, a most august feature in the physiognomy of a country."

"The climate, too, of New-Orleans must not be overlooked, as its peculiarity, co-operating with the above-mentioned distribution of the Mississippi and the condition of the soil, is the real and only cause of those formidable diseases to which this city and its its vicinity are subject. It is one of the anomalies of the New World, not yet very satisfactorily accounted for, that the intensity of the heat in summer, and of the cold in winter, is much greater than in the Old World, on the same parallels of latitude. This difference is very obvious all along the eastern shores of the American continent, but nowhere is it so striking as at New-Orleans. From the end of November till the end of March, the weather is generally cold and rainy, with frequent hard frosts. At those times the thermometer ranges from 20° to 40° in the shade; and there are instances, I am told, even in so low a latitude as 29° north, where, in the night, it is only a few degrees above Zero. On the contrary, during summer this climate has all the characteristics of the torrid zone; the thermometer stands at 87° or 90° in the shade. At New-Orleans, especially, the weather is close and suffocating, from its distance from the sea, and, consequently, the entire absence of that inestimable luxury of a tropical climate, the sea-breeze; from the air being loaded with watery vapours; and from the smell of the mud of the river and swamps, which is often, even in winter, very sensibly offensive."

"The local peculiarities in the climate and soil of New-Orleans give rise, during winter, to epidemic dysentery, and, in summer, to marsh fevers of a very rapid and dangerous form, from which the inhabitants, but particularly strangers, suffer most severely. The occurrence of such complaints, some readers, from the above detail, will be prepared to expect as a necessary consequence. Lest others, however, should be sceptical, it shall be my business, by and bye, to make this preliminary picture of the medical topography of the country subservient to discussions of higher interest, and to prove, by facts, the reasonableness of opinions.

"'Hoc opus exiguum *vario sermone* levemus,
Perque vices aliquid quod tempora longa videri
Non sinat; in medium vacuas referamus ad aures.'
Ovid. *Metam.*"

We have selected this short passage, not as containing any thing very new, but as forming a fair specimen of this naval surgeon's descriptive style. We shall now confine ourselves to the diseases and their treatment.

"About the beginning of January (1815), bowel-complaints, which had previously appeared amongst the boats' crews and the fatigue-parties of the army, began to be very rife. They varied in degree of severity, from the milder symptoms of dysentery to its most aggravated forms. I may enumerate, in a few words, the symptoms of this disease. The patients, for the most part, complained of severe tormina, tenesmus, scanty bloody dejections, want of appetite and strength, pains all over them, and a disposition to vomit on taking either food or drink. The tongue was white or yellow; the eye languid; the pulse above 100, small, and easily compressed; compressed; the skin often dry, or covered with clammy sweat, but always considerably increased in temperature.

"The causes were, generally speaking, obvious enough. The men had been rowing all day, and sleeping all night in the open boats. They had incautiously drank the brackish water of the lakes, and had sometimes been obliged to eat their beef and pork raw, when, on an emergency, they were deprived of an opportunity of cooking it. They were often drenched with rain or with spray, without being able to put on dry clothes. Added to all this, the weather was extremely cold, particularly in the night, the thermometer before sun-rise being often as low as 25 or 26 degrees, rising no higher during the day than 30 or 38 degrees, and seldom above 50 degrees. These observations of the thermometer are repeated from my own inspection. On this occasion the cold was so intense, that several of the boats' crews were incapacitated for some days, by pain and numbness of the lower extremities. Many soldiers of the negro regiments had their feet frost-bitten, and lost their toes by the consequent gangrene and sphacelus. Some of them even died in the camp, or in the boats, from excessive cold."

Nothing particular occurs in the practice, which began with purges, was continued with small doses of ipecacuanha, and concluded with the several means of restoring health or assisting convalescence. In the severer cases, the author very judiciously added the use of the lancet, and we can heartily believe him when he says that he never had occasion to repent the recourse, however frequent, to that remedy. On the subject of opium, we have nothing to say against matters of fact; but experience has taught us to avoid it till all inflammatory symptoms seemed subdued. We conceive the same may be said of injections, which, as the author observes, almost the whole body of the profession have concurred in praising. If given during the inflammatory state, or at the close of hopeless cases, "the irritation occasioned by the bare introduction of the instrument, more than counterbalances any advantage." We can add, that, if that is surmounted, the irritation from the fluid injected, however bland, adds to the distress of the author. But in an advanced chronic state of the disease, we have found all the advantages promised by other authors; and even after opiates, testaceæ and ipecac. have failed. We are, however, much pleased with the author's suppositories of very small portions of opium, and with his fomentations applied to the parts, and bladders of water to the abdomen.

We shall pass over the military account of the expedition, not, however, without remarking the pleasure we felt at what we are willing to call the juvenile ardour with which the assault on Troy and the streights of Thermopylæ are introduced, troduced, with the sage discretion of Falstaff applied to the Americans, and, last of all, "the envious wall which stood betwixt them and the approach of British vengeance.

"*Invide* dicebant, *paries*, quid *fortibus* obstas? -

Quantum erat, ut sineres nos *toto corpore* jungi!!"

OVID. *Metam.*

"This sentiment burst indignantly and unanimously from our troops."

As none but the brave deserve the fair, the change of *amantibus* for *fortibus* may be admissible as a pun; but, often as we have heard of love and war, or the junction of Mars with Venus we were not prepared for the whinings of Pyramus and Thisbe on such an occasion.

Some remarks follow on the comparative healthiness of the troops during active service, and their subsequent relapse into ill health. Here, we conceive, our author imputes too little to change of season, the siege and assault being in the winter. As the warm weather advanced, he observes,

"Dysentery now put on that exasperated form in which it has so often scourged our camps and fleets; and never shall I forget the terrible force of this invisible enemy. In all cases it was a very baffling untractable disease, but in those who had previously served long in warm climates, and whose livers were thereby affected, it was almost uniformly mortal. When the disease attacked such persons, it was a subject of melancholy but curious speculation to witness the headlong course of the disease, and how unavailing any species of treatment invariably proved. It knew neither pause nor hindrance, but, like the fabled vulture of Prometheus, pursued its cruel task from day to day. Dissection always brought to light extensive visceral obstructions, particularly chronic inflammation, or abscess of the liver, with or without enlargement.

"Nothing but experience can convey adequate ideas of the ungovernable nature of this disease, or of the insidious masked approaches of its attack. Days of an indisposition, apparently trivial, sometimes occurred ere the peculiar symptoms of dysentery shewed themselves; at other times, pyrexia, high or slight,* and occasionally pain in the right side, obtuse or acute, followed by frequent copious dark green stools (like *boiled spinage chopt*), slightly tinged with blood, were the form of the disease.

"* I may observe, that I never had the slightest reason to believe the disease itself, or its attendant pyrexia, to be at all contagious. I may also remark here, though I anticipate the course of the narrative, that in April and May, when the weather became hot, the character of the prevailing dysentery was rather exasperated by it; unlike the dysentery of cold climates, which is generally rendered milder, if not extinguished, by atmospheric heat.

"Griping was little complained of. There was merely a sense of weight in the hypogastric region, and a copious *flux* of green or dark-coloured sordes, voided without straining. The tongue was covered with a yellow fur, which, in the advanced stage of the disease, became thick, dark, and immoveable, as a slab of black marble. The pulse was sharp, but weak; frequent retching and hiccup attended; and a sensation, as if all the drink swallowed, hot or cold, ran speedily through the intestines. Oftener the complaint would make its attack with the common introductory symptoms, and no pain in the right hypochondrium was felt throughout the disease, either on inspiration, or strong pressure beneath the ribs. Under whatever garb of disguise it made its appearance, disease of the liver (as I have before stated), and consequently a vitiated state of its secretions, were undoubtedly the primary cause of the mischief. Dissection of the fatal cases shewed structural derangement, and generally suppuration of that viscus. I have often found two separate abscesses in the central part of its large lobe, containing in some instances a pint of pus, similar in colour and consistence to what is usually found in psoas abscesses.

"On the villous coats of the colon and rectum, there were numerous excoriated points, with small superficial ulcers here and there, like the sequelæ of erythematous inflammation; but there were no morbid alterations sufficient to account for death; no ravages of gangrene, &c. like those related by Sir John Pringle and others, in their accounts of this malady.

"In short, to give a condensed view of the whole matter, the phenomena of the cases that recovered, as well as the morbid appearances of those that died, impressed upon my mind a conviction, that the diseased condition of the liver was the soil from which dysentery drew its malignant growth, strength, and nurture. This was the 'fons et origo mali;' by it the dysentery was excited, and only by *its* removal could it be removed. This double detriment—this *Janus-like* aspect of the disease, I rather think, is new to many of the profession, but I trust it will soon be widely known and acknowledged. I hope the time is not far distant, when, instead of viewing dysentery as an idiopathic disease, and tracing its seat to the colon and rectum, medical men will regard it merely as secondary to, and symptomatic of hepatic affection, and will seek its cause in a morbid condition of that important gland.* Whatever may be the mode of connection between hepatitis and dysentery,

"* It seems to be one of the errors of modern medicine, to overlook in practice the liver and spleen, merely because the necessity of their functions is not so obvious and immediate as that of some other organs. That a gland so large and of such unexampled vascular structure as the liver, should have much occult influence in all diseases, might, from the mere reason of the thing, be supposed. Its secretions influence the state of the stomach, and are tery, I have no doubt that, at least in tropical climates, they are connected like cause and effect. I am unwilling to offer any hypothesis on this subject, purely because I am unable; this I confess, for I shall never chime in with that tone of affected contempt for all theories, in which presumptuous dulness so often shelters its imbecility. Those who indulge this disgusting oft-repeated cant, ("crambe bis millies cocta,") ought to be reminded, that not merely in medicine, but in all other sciences, few brilliant discoveries have been made, except by those acute and industrious men that were shapening and toiling at some untractable theory. However much all their diligence might fall short of the results they themselves fondly expected, still so much digging and delving often turned up very valuable ore, and always left the soil in a fitter state for the future labourers in the great field of improvement.

"To return to the subject under consideration, I can readily conceive, that, from disease of any gland, the fluid it secretes may acquire acrimonious properties, sufficient to injure the fabric of the passages through which it is destined to pass. We generally observe in dyspeptic complaints, or after a period of constipation, when the bile, from *remora* in the bowels, becomes morbid in quantity or quality, either that a spontaneous diarrhœa comes on, or, after a brisk cathartic has been exhibited, that the dislodged bile excites a sensation in the rectum, as if boiling lead were voided. When the state of the liver is still more morbid, may not the bile acquire the property of exciting flux, and of excoriating and ulcerating the villous coat of the colon and rectum?"

Some apologies follow for offering this theory, and the

---

are influenced in their turn by the passions of the mind; and many facts would lead us to believe that there is a hitherto undescribed sympathy betwixt this viscus and the brain. I am informed, from a gentleman who has practised long in India, that patients have been suddenly seized with amentia, rigors, delirium, and syncope, speedily followed by death, and that, on dissection, abscess of the liver was the only perceptible cause of such symptoms.

"The depressing passions I have seen to have a striking effect on the biliary secretion, and even to induce cholera; whereas anger, like intoxication, when habitually indulged, gives rise to chronic enlargement and obstruction of the liver. In short, the functions and sympathies of this gland, which were deservedly of so high account with the ancients, seem to be insufficiently studied by modern physicians.

"Horace, in the following lines, instead of a popular or poetical tenet, has probably expressed a curious and unexpected pathological fact.

"'———————— Vae meum

Fervens *difficili bile tumet jecur*

Tunc nec *mens* mihi, nec *color*

Certâ sede manet.'"—Horat. *Carm. Od.*

 paragraph paragraph closes with a sentence from Cicero, which has been quoted pretty often by others.

The respect we owe this writer, induces us to offer him a remark which we are certain will not be thrown away. We have no doubt of the *origo mali;* but some doubt of the disease itself. Thee vacuations resembling "boiled spinage chopt," the want of griping, and still more of straining, would have induced us to describe these evacuations as proceeding from a diseased liver, and not from the inflamed or ulcerated state of the mucous membrane of any part of the intestines, the proximate cause of dysentery. As to the remedies, we shall only remark,, that, however judiciously calomel may have been given, we suspect the writer will hardly conceive it equal to the cure of abscess in the liver, or of other diseased structures which he discovered in his dissections. We suspect also that it did not prevent them, as the remedy was probably not exhibited till the disease was formed. Whether it occurred to the author late in the season, when none but the milder cases had survived, we shall leave to his recollection.

After the freedom of this remark, it becomes us to add, that in a subsequent paragraph the author is not less candid in acquainting us with unsuccessful cases under the use of calomel, than he was before sanguine in recommending it.

Some very judicious observations follow, but not very new, on the effect of scurvy.

We were much pleased with the facility with which cholera is dismissed,—in our opinion more commonly an action for the preservation of health than of a threatening aspect. Still more pleased were we with his manly objection to the whimsical system of nosology, which has not only burthened the memory of students for the last half century, but misled their judgment. Speaking of yellow fever,

"That cabalistical word *typhus,* I verily believe, has slain its thousands and its tens of thousands. The effect of a mere word is often prodigious; for, as the famous Mirabeau once said in the French National Assembly, 'words are things.' Terms signify ideas,—these constitute opinions,—and opinions lead to acts. Every body is now convinced how improperly the term Typhus is affixed to the endemic fever of the West-Indies; that it is applied with more propriety to the majority of fevers in our own country, is to me by no means clear. While I acknowledge that, in the *made-up* constitutions of artificial life,—amidst the squalid dregs of the population of a crowded and high-viced metropolis,—some cases of fever occur where the brain labours merely through sympathy with the stomach and biliary organs, and where the lancet, for several reasons, is unnecessary, or inadmissible; still, in by far the greater part, I suspect the reaction is sufficiently violent, and the the determination to the contents of the head and belly sufficiently marked, to require, and to be greatly benefited by, blood-letting, either general or topical, or both. The fever, however, is apt to be hastily pronounced typhus, and, this sentence once passed upon it, typhus it must be; consequently, from day to day, the name of a disease is prescribed for with due solemnity and skill. To be sure, the morbid actions are fortunately seldom so concentrated as to resist the subordinate evacuations of purging and blistering; and so the patient frequently recovers in the end, after a protracted illness, which he, 'good easy man,' thinks has been quite unavoidable; charitably supposing 'all's well that ends well.' Even if we keep out of view the high moral responsibility for risks run, and sufferings protracted, which this inert treatment implies,—even if we speak of it with mildness,—we cannot, in conscience, bestow on it any other than the negative commendation, that by its effects neither the patient loses his life nor the practitioner his reputation!

"The same erroneous nomenclature which gave to ardent fever a typhoid character, in all likelihood originally produced the notion of its being contagious,—a notion which has since been attempted to be maintained by a combination of learning and sedulous talent, that, by plausible reasonings and expertly laying hold of popular opinion, has sometimes had power to 'make the worse appear the better reason.' But the affinity which such nosological arrangements suppose, does not hold. Besides the known fact that febrile contagion will not exist in warm climates, but is more readily extinguished by atmospheric heat than by any other cause, there is such a difference in the first symptoms, progress, and duration, of ardent fever from those of typhus, that all who are guided by practical views, and are not misled by too eager a spirit of generalizing, have pronounced it a totally different disease,—in fact, a disease of inflammation. Such a radical difference of character argues a corresponding difference of causation. The origin of this fever has, therefore, been attributed to causes of a local or domestic nature, because the disease itself is found to be strictly local. It only prevails in countries within the tropics, and in them only at those seasons when the thermometer ranges from 80° to 94° in the shade. It is, therefore, justly believed to be owing to the diffusion in the atmosphere of those poisonous exhalations, which are elicited by the powerful rays of a vertical sun, from marshes, from putrefying vegetable matter, or from the soil itself of tropical countries. Miasmal poison is one of the most widely-diffused causes of disease throughout the whole province of nature; and if northern climates know less of its pernicious effects, they owe this happy exemption solely to the inferior power of the sun's heat in collecting those noxious vapours.

"Although the disease I speak of may be said to have its seat and throne within the tropics, yet in every country where the height of the thermometer is at certain seasons from 80° to 90°, fever, instead of the low type observed in high northern latitudes, assumes, assumes, in almost every instance, a decided inflammatory character. In short, amongst the febrile diseases of southern climates, there is a uniformity of character, which, in spite of hypothetical classification, powerfully argues a community of origin and of cause. For proof of these facts, I may refer to the valuable practical works of Dr. Irvine on the Diseases of Sicily, and Dr. Burnett on the Fever of the Mediterranean; as also to a judicious paper by Mr. Boyle, published in the 6th volume of the Edinburgh Medical and Surgical Journal; and to the various reports of the American physicians.

"Thus, New-Orleans, though without the tropics, is almost every summer visited by a *four* or *five-day* fever, which has all the essential characteristics of the genuine *kausus*, and is, in fact, known popularly there by the name of yellow-fever. This heavy infliction is entirely owing to its climate and locality, (which I have already taken pains to describe),—to that profusion of marshes with which it is surrounded."

In another passage the author has the following words in a note appended to this word *kausus:*—"Why is this word usually spelt with an initial C, since we all know it is derived from the aorist of the verb καιω, καυσω, κεκαυκα, uro?" We are surprised at this prudery in one who has given so many proofs of scholarship. Is it not almost an universal change, adopted, probably, from the Latin, whose language did not possess legitimately a K, and whose C had probably always a sharp pronunciation. But the same objection might be made against anasarca, cynanche, scirrhus, and several other words.

We have given, though a long, by no means a full, account of this valuable paper, which, we trust, the author will enlarge into a pamphlet. He must not, however, suppose that his suspicions of the inflammatory nature of our northern fevers originate with himself, nor even his strong objections to the word *typhus*. Dr. Jackson's persecution originated in his free bleeding of patients whom he cured; and Dr. Sutton most amply confirmed this practice. Though the term typhus was not in use in Sydenham's time, we find him not less severe against those who were always apprehending malignity.* The first objection we recollect against nosology in general, and more particularly against the unfortunate word *typhus*, is in Adams on Morbid Poisons. "To this," says Dr. A., "by an easy transfer, was added the term putrid fever, and afterwards TYPHUS was re-echoed from the Professor's chair to the nursery," &c.† In short, the remainder of this paper seems almost a transcript of Dr.

* Scedula Monitoria, parag. 41.

† Adams on Morbid Poisons, 2d edit. p. 375.

Jackson's

Jackson's practice, and of Dr. Adams's opinion concerning contagion.

We cannot fail to offer an extract on the important question concerning the cure of fevers by calomel. If bleeding and other evacuations are sufficiently attended to in the beginning, it is, perhaps, of little consequence what the subsequent treatment may be; but a dependance on any other remedy always endangers a feeble practice, inasmuch as it furnishes the practitioner with an apology for yielding to his timidity.

"These remedies are mentioned in succession according to their relative efficiency, but, in actual practice, their application must be contemporaneous. Bleeding, purging, cold lotions to the head, shaving the scalp, and general refrigeration by the cold bath, must be drawn up together in array against the disease, and must make a combined attack. A first or even a second disappointment must not rob us of our perseverance. Courage and constancy will, in the end, often succeed against great seeming odds. In short, the violent excitement must be got under by all means, ordinary and extraordinary.

"I have never either tried or trusted to calomel as a *sialagogue* in this disease. The blind confidence in its supposed specific power has, I believe, nearly faded away before the better lights and the more speedy results which the depletory practice has afforded.* In ardent fever, where there is a morbid activity of the arterial, with a proportional inactivity (almost amounting to torpor) of the venous and absorbent systems, it is a matter of extreme uncertainty whether mercurials can find their way into the system until the paroxysm of fever is dissolved. Its action, even were it absorbed, would be rather hurtful, as favouring that deprivation of the solids, and solution of the fluids, which, with the effect—putrescency, are so much to be feared in the latter end of continued fevers. Upon the whole, longer time and trials have only given additional strength to the opinion which Dr. Saunders pronounced on the inutility of mercury in the endemial fevers of tropical countries."

"* An attempt has lately been made to *clap up a match* betwixt the depletory and mercurial methods, and to call in the aid of both in the same case. The most respectable, if not the original, proposer of this incongruous union, is Mr. Johnson, in his valuable work before referred to (see note, p. 136). What table of affinities suggested this coalition, it would be vain to conjecture.

"However ingeniously devised this combined system may be, it will never stand. Like the famous image in the vision of the prophet Daniel, it is formed of repulsive materials: the *iron* and *clay* will not coalesce—cannot amalgamate—but the baser matter will crumble to dust, leaving the other part to the enjoyment of proud perpetuity. The separation, doubtless, will be spontaneous, and the sooner it takes place the better."

We believe Dr. Saunders confines his objection to the western hemisphere. In the East the use of mercury is not disputed, though even here it is now admitted by able practitioners, that, where the symptoms are urgent, bleeding is often necessary before mercury can produce any effect on the disease, or its specific action on the mouth.

Such are the outlines of this ingenious paper. The conclusion, though not strictly medical, will be read with much satisfaction by all, as a well-deserved encomium on our countrymen. It appears to have been written immediately after the battle of Waterloo, and that event is happily introduced as the climax to a long series of heroic exploits.

[The remainder of the articles in our next.]

---

*Medico-Chirurgical Transactions, published by the Medical and Chirurgical Society of London.* Vol. VI.

(Continued from p. 328.)

*A Case of Lock Jaw, cured by Oil of Turpentine given as a Clyster;* by EDWARD PHILLIPS, M.D.

THIS case is well worth being recorded. We think, however, the title somewhat too decided in its language. The subject was, as the writer informs with great correctness, "of a delicate and sensitive habit, always much affected with slight mental irritation;" hysterical also. In these subjects it is not always easy to form a correct judgment of spasmodic diseases. It is, however, no slight consideration, that a turpentine clyster removed so many tiresome, if not formidable, symptoms in such a subject.

*Case of an extraordinary Enlargement of the Scrotum, with an Operation successfully performed for its Removal.* By JOHN MADDOX TITLEY, M.D. of St. Christopher's.—(See our COLLECTANEA.)

*On the Use of Nicotiana, in Retention of Urine.* By HENRY EARLE, Esq. Surgeon to the Foundling Hospital.

The first subject whose case is here related,

"When about eighteen years of age, had suffered severely from gonorrhœa and hernia humoralis; from this period he dated the complaint in his urethra. He was now thirty-five: during this time he had been in a gentleman's service as groom, and had been obliged to ride a great deal. The stream of water gradually diminished in size, accompanied with frequent and urgent calls, until about two years before the present period, when, from being obliged to remain a long time on horseback, he had a retention of urine, accompanied with so much inflammation, that an abscess formed in the perineum, which burst and became fistulous. For this complaint he had been for some time under a surgeon's care, who attempted to pass bougies, but never succeeded in reaching the the bladder. He had latterly been in the habit of passing a metallic bougie for himself, which was the probable cause of the present retention and inflammation.

"On examination, I found a firm obscurely elastic tumour, about the size of a pigeon's egg, situated immediately on the urethra, at the lower part of the scrotum. This was about the point to which he had been accustomed to pass the instrument. The surrounding scrotum was healthy, which led me to refer the present abscess rather to the irritation of the bougie, than to any effusion of urine, which generally diffuses itself more extensively. The abscess had been about three days in forming, accompanied by great pain and fever, and he had not been able to void his urine for the last eighteen hours. I immediately made a free incision into the abscess, and let out about ℥iv. of very fetid pus. I directed him to sit in warm water, and ordered a common clyster to be thrown up. As he was still unable to make water after the trial of these means, I desired him to take fifteen drops of tinctura ferri muriatis, every ten minutes, in barley water. He continued it for nearly three hours; the medicine produced nausea and head-ache, but still no water passed. I now attempted to introduce a bougie, but could not get beyond six inches; the introduction thus far was productive of great pain. His symptoms were now very urgent, for, although the bladder was not greatly distended, yet from the long existence of disease, it had probably become much thickened, and was very irritable. Apparently no alternative now remained but an operation; and, as the bladder could not be satisfactorily felt above the pubes, and the perineum was much thickened and diseased, I determined in my own mind to puncture from the rectum. Previous, however, to resorting to this *ultimum remedium*, I was desirous of trying the effect of the nicotiana."

The proportion of tobacco was an infusion of two drams to a pint of boiling water; half was thrown up, and with great difficulty retained. In ten minutes the patient felt alarming symptoms of intermitting pulse, clammy sweats, extreme sickness, and faintness; the urine escaped by dribbling. The contents of the rectum were suffered to escape. The patient continued mending in all his complaints till he was obliged to leave Mr. Earle, on account of engagements in the country.

The next case was one of strictures in their worst form. After repeated attempts with the common bougie, Mr. Earle applied the lunar caustic. In the midst of his attempts, the patient was seized with so severe a retention of urine, as induced the application of nicotiana. The infusion was thrown up as in the other case, with much less constitutional disturbance, but with still greater success.

A third case follows, equally successful, and attended with similar circumstances. From all which we may conclude, that under violent spasms much is to be expected from this valuable, but not less serious, remedy. The whole, however, does great credit to Mr. Earle.

A very interesting case of tetanus follows, with the dissection. Nicotiana seemed to alleviate the symptoms; and bleeding, which in the course of six days was carried to the loss of nearly seventy ounces, still more so.

"On Sunday (says Mr. Earle) he was so much better that I entertained confident hopes of his surviving; the principal pain he complained of was across his back and belly, and in one thigh. On Monday he continued to improve, but unfortunately his friends paid him another visit, and at his request gave him some porter, which reproduced the spasms, but in a much diminished degree. Towards evening he complained of weakness, his countenance had changed much during the day, his pulse became very frequent, and when sleeping he rambled a little. During the night he sunk rapidly, and the following morning expired. The blood, to the very last, exhibited the strongest marks of inflammation.

"*Dissection.*—Nothing particular was found in the head; the veins of the pia mater were rather turgid with blood. The thoracic and abdominal viscera were healthy. On the surface of each psoas magnus muscle there was an effusion of blood, on removing which the muscular fibres were seen much lacerated and altered in texture, being quite soft, and giving way under the fingers. This lacerated appearance occupied nearly the whole thickness of the muscles to some extent. A similar effusion of blood had taken place in the sheath of the right rectus abdominis, and the muscular fibres were torn and quite soft, like the muscles of an animal that had been hunted to death.

"Though this case terminated fatally, I conceive that the appearance of the blood and the remission of pain on bleeding, fully warranted my prosecuting this plan. The muscular fibres being actually torn asunder, sufficiently prove the violence of the attack; and I think that although I was not so fortunate as to preserve his life, I rendered his severe sufferings more supportable. Blood-letting, in tetanus, has been much censured by different authors, but I must hesitate in subscribing to their opinions, which I conceive have been formed without sufficient trial, or rather, which have been adopted from one generation to another, without the acquisition of any new facts. I am induced to form this conclusion from meeting with the following passage in Cullen. He says, 'Blood has often been drawn in this disease, but it never exhibits any inflammatory crust, and all accounts seem to agree that the blood drawn seems of a looser texture than ordinary, and that it does not coagulate in the usual manner.'

"Now either the case which I have just related was anomalous, or Cullen has adopted opinions without sufficient grounds. As I do not possess any other facts on this head, I cannot pretend to decide on this subject, but scruple not to delare, that should such a case again occur, with as much arterial action, and with such appearances of the blood, I should certainly adopt a similar line of practice, combined with the use of nicotiana."

It surprizes us greatly, that so many well-informed surgeons of the present day, should omit to mention the time after death when the examination of the body took place. It would be particularly interesting to know whether, immediately after death, the muscles, those of the heart particularly, were rigid, and afterwards became soft; as such was the case with Mr. Howship's invaluable case contained in our Journal, and to which we have so often referred.

*Case of Incontinence of Urine of Nine Years standing, cured by Pressure.* By John Hyslop, Esq.

This is a very interesting case. The pressure was accomplished by a large bougie applied on the outside of the urethra, and fastened by straps of adhesive plaister through the whole length of the urethra. The success was much quicker than could have been expected. The author concludes with proposing a similar application in cases of paralysis, for the purpose of cleanliness, if not of cure.

*A Case of Aneurism, by Anastomosis in the left Orbit; cured by tying the common trunk of the left Carotid.* By W. Dalrymple, Surgeon, &c.

This is an interesting case, but cannot be shortened. From the recurrence of two hæmorrhages, and the complete recovery of the patient after the last, we would again suggest what we have often urged, the propriety of bleeding a patient freely when marks of inflammation occur after an operation; and even of suspecting that the unfavourable appearance of any wound, at one time going on well, should lead us to such a suspicion, especially if that unfavourable appearance has commenced with rigor, and been followed by serous discharges.

*Account of a Case in which parts of a Fœtus were found in a Tumour situated in the Abdomen of a Girl, two years and a half old.* By Edward Phillips, M.D. &c.

The subject was born to all appearance healthy, and no abdominal enlargement perceived till the third month. From that time the abdomen enlarged, without materially affecting the child's health, till about two months before death. On examination after death, a tumor was discovered, extending from the edge of the diaphragm nearly to the pelvis, and adhering firmly only to the left kidney.

"The tumour, when removed from the body, might have weighed about eight or ten pounds. It was of an oblong shape, loosely covered by a delicate membrane, highly vascular. On making a section of it, some ounces of a limpid fluid escaped from a cavity, the parieties of which were nearly cartilaginous; and, in prosecuting the dissection, several similar compartments were discovered, all of which contained fluid or sanious matter.

"In the further examination of this fleshy mass, our attention was arrested by the resistance which the knife met with, and which led to the discovery of the bones which I have sent you. They were connected to the internal substance of the tumour by a structure decidedly muscular. The large bone, resembling the tibia, was covered by muscle; the small bones, resembling those of the tarsus, were connected to the tibia by soft cartilaginous bands."

This is certainly a most interesting case, but we should not have been sufficiently bold to call it a *fœtus* in a child. The various substances of which the tumour was made up, rather impresses us with the idea of a process similar to hairs, teeth, and other substances, found in various parts of the body. If these more commonly appear about the appendages to the uterus, the same may be said of hydatids, and other incysted tumours.

*Case of Axillary Aneurism, for which the Artery was tied below the Clavicle.* By RICHARD CHAMBERLAINE, Jun. Esq. of Kingston, in Jamaica.

This aneurism was the effect of an accidental wound: the vessels were therefore probably healthy. The operation is so extremely interesting, that we shall give it in the words of the author. It was performed between three and four months after the accident.

"The patient was placed upon an operating table, with a pillow under his shoulders, and his head supported. A transverse incision, of three inches in length, was made through the skin and platysma myoides, along and upon the lower edge of the clavicle, three fingers' breadth from the sternal extremity of that bone, and terminating about an inch from the acromion scapulæ. This incision divided a small artery, which was immediately secured. A second incision, of three inches in length, was also made obliquely through the integuments over the deltoid and pectoral muscles, meeting the first nearly in the centre. The cellular membrane, and fat lying between them at the upper part, were now removed. The next step in the operation consisted in detaching the clavicular portion of the pectoralis major, and taking away the fat and cellular membrane lying over the subclavian vessels. The artery was now brought into view, and its pulsations made it clearly distinguishable from the contiguous parts; but I could not detach it, nor pass the ligature underneath it with the facility I expected expected, from its depth. After several ineffectual efforts, I succeeded in conveying the ligature under it, by means of an eyed-ball probe, previously curved for the purpose, and bringing up its point with a pair of forceps, tied the artery as it emerges from under the clavicle to proceed to the axilla. The drawing of the knot was attended with little pain; the wound was closed by the dry suture, and the patient returned to his bed.

"*Evening visit after the operation.*—Pulse natural; skin cool; affected arm appears much warmer than the other; complains of pain in the wound."

This pain continued for a considerable time, and the heat also. As the case turned out so very favourable, it may seem invidious even to enquire whether the pain might not have been relieved by bleeding. The operation, however, and subsequent treatment, does great credit to Mr. Chamberlaine. The author regrets that he was not furnished with the instruments described by Mr. Ramsden.

"February 22, (thirty-six days after the operation,) the wound healed; the aneurismal tumour is now nearly as large as a turkey's egg, and very solid, but no pain is felt when it is roughly handled. Upon comparing the arms, the left is rather smaller; the muscular power of the arm is much improved, inasmuch as he now grasps with a greater degree of firmness and strength; temperature of the arms is the same; discontinued to visit him.—N.B. The temperature of the axilla was always ascertained by a well graduated thermometer."

*Case of Cynanche Laryngea*; by James Watson Roberts, of Bishop's Stortford.

This and the succeeding paper are highly interesting and important, not only on account of the formidable disease described, but the accuracy of the description, the successful treatment, and the ingenious, candid, and learned remarks of one whom, not only the public voice, but the opinion of the profession, has raised to the highest rank in the metropolis.

The subject of the case was the army-physician who a few years after died of the same disease. The description, by Dr. Roberts, is so truly that of inflammation and congestion about the head and throat, that we can only wonder any doubt could be entertained concerning the propriety of free bleeding. In the present day we may wonder still more, as some of the symptoms were analogous to the yellow fever. We must, however, reflect that the event happened in the year 1794, when the opinions of the meritorious, but ill treated, Dr. Jackson, were ill received by those who should have known better. Another objection to bleeding, equally unfounded, was the apprehension of a gouty gouty habit; but the most unfortunate of all was, the suspicion of spasm. Oh! the influence of words! At one time they frighten us more in proportion; as, like supposed aërial beings, they have no real meaning affixed to them, and consequently the imagination supplies every thing terrific. Thus typhus, and its associate, typhus icterodes, has thinned our armies and fleets more than the sword, or even the tropical sun. At other times words impose a law on our practice, not less absurd than those of the Medes and Persians; which cannot, under any circumstances, be reversed. We even forget our law-giver; for Sydenham, who first objected to bleeding in gout, only confines his interdiction to chronic gout, often advising the lancet in the acute. But most dangerous of all are those words which serve as a cover for ignorance or timidity, and for this purpose nothing is so general as Spasm.

Having made these remarks, we shall return to the author of the paper. Unawed by the wise shrugs of ignorant systematics, or even by their dereliction of the patient, he used the lancet at first indeed with becoming caution, from his respect to his brethren; but, gaining courage from success, and no longer awed by the fears of others, he succeeded at length in carrying his patient through this formidable disease.

The remarks by the President, in the subsequent paper, are equally learned, pointed, judicious, and candid. We can, therefore, object to nothing; but the leisure of us who are doomed to write, may enable us to produce more authorities than one so constantly engaged in practice.

First of all we cannot help recurring to a subject we have so often mentioned, we mean our objection to nosology. To what but nosology do we owe all our errors of treating fevers from the apprehension of typhus. To what but nosology do we owe the error we have just hinted, in treating gout by its name and not by its symptoms.

"As nosology, (says the learned president,) was not in use as one of the methods of cultivating physic till our times; as there is no where to be found, before this invention, any catalogue of all existing diseases; and, as no author professes to treat of all known diseases, we have no means of ascertaining precisely what diseases existed and what did not, at any particular period. Works of nosology, therefore, even though founded on a vicious system of arrangement, possess great value merely as comprehensive catalogues of diseases, and as affording scope for accurate description and discrimination.

"The infrequency of this disorder seems to be a main reason for its having been so little noticed, and it seems to be a wise provision vision of nature that it should be so rare. If the glottis were by nature as liable to inflammation as the tonsils, the human species would have difficulty in maintaining its existence.

"We have, in the present case, an example of its real nature having been overlooked, for the first seizure passed at the time for the modification of a common sore throat. The casual coincidence of two eminent physicians in the metropolis having been affected with it about the same time, has led to an investigation which has established its specific nature. We seem now, therefore, to have a very complete nosology of the diseases of the throat; for the cynanche tonsillaris,* the most common species of sore throat; the C. Pharyngea, or quinsy; the C. Parotidea, or mumps; the C. Trachealis, or croup; and the C. Laryngea,† now under consideration, admit of descriptions as specifically defined as any objects of natural history.

"The number of cases of this last species (the most rare of them all) which have already found a place in our Transactions, affords a striking proof of the high value of such ample repositories of facts brought so closely and rapidly together, as to throw the most interesting and instructive light upon each other. When single cases of any disease, particularly a rare one, lie scattered in different works and at long intervals of time, the advantage of comparison and induction is in a great measure lost to the practitioner, who has to decide promptly on his measures; and it is hoped that the advantageous manner in which these cases have been brought forward, will lead to a more successful treatment in future, for it is a melancholy and mortifying truth, that of these eight cases six have terminated fatally."

When we meet with such a passage from one almost born in a dissecting room, educated in hospitals, and above all, who has devoted his whole life to a profession in which, for more than twenty years past, he has been consulted in most acute cases; a variety of reflections occur, but the most

* "The ancients do not include this under the appellation of *Cynanche*, or *Angina*. A difficulty of respiration, agreeably to the etymology of these words, was an essential character of them. The Greek and Latin authors call the C. Tonsillaris, merely an ulceration of the tonsils. They would, for the same reason, have excluded the C. Parotidea from this genus; but there is no allusion to this disease in any ancient author that I know of.

† "There is a variety of the C. Laryngea, of which three cases have come under my observation. It consists in a state of chronic inflammation and suppuration of the larynx. They all proved fatal; and, two of them being inspected after death, *pus* was found in all the interstices of the muscles, bones, and ligaments, and the organization of the whole considerably impaired. From the similarity of the symptoms, I inferred the third to be of the same nature."

striking striking of all is, that there must be some revolution in the diseases of mankind, dependent, as far as we can judge, on what Sydenham imputed to the constitution of the air or season.

We shall first, therefore, show that an inflammatory disease, requiring similar remedies, and equally dangerous if those remedies are not promptly applied, was well known to the ancients and to Sydenham; and next, endeavour to maintain our opinion, that nosology, if not injurious, is at least so in many of the discriminations above alluded to. Angina was the term generally applied to it. The Greeks, who were always fond of compound words, used the term Συναγχη, which Aretæus considers a corruption of Κυναγχη, and derives it from the frequency with which dogs seem to breathe with difficulty. Celsus refers to the divisions made by the Greek writers; but, according to his accustomed brevity, enumerating nothing more than is absolutely necessary. From this little we learn, that the disease was considered serious in proportion as the parts in sight were free from inflammation or swelling. In these cases he considers the danger not less than in tetanus, and advises blood-letting, though the patient should not be plethoric. His caution, *si vires patiuntur*, probably refers to the more advanced stage of the disease, in which nothing can be done. He proposes also cupping under the chin, and about the fauces; and afterwards adds the application of hot salt in bags, as a most efficacious remedy. There is nothing in this that ought to be overlooked in our days.

Aretæus is not less alarmed at a disease, the sudden effect of which he compares to the pestilential vapours arising from groves or caverns, or even to poison. Both these writers are equally careful in distinguishing this disease from inflammation in the anterior parts of the internal throat, or posterior parts of the mouth, as the uvula and tonsils. Vansweiten informs us (we have not taken the trouble to examine for ourselves) that Aegineta makes a division of cynanche, paracynanche, synanche, and parasynanche. All of these writers describe one form of the disease as particularly fatal.

But we need go no further than Sydenham to trace the rapid and dangerous progress of angina. He gives, indeed, no nosological division, considering the inflammation as attacking every part of the throat; but instances the larynx, and describes the danger of suffocation. His practice is such as has been so judiciously recommended by the author of the paper, viz. early, free, and repeated bleeding and purging. In his monita he adds a large strong blister-plaster, not immediately immediately over the part affected, but to the back. It is not a little remarkable that Sydenham should propose bleeding the vessels under the tongue, which makes it probable that he had read Aretæus, though he rarely brings forward his learning, unless to defend his practice.

Having made these free remarks, we shall conclude with our former observation, that there seems something in the constitution of the air which produces its effect on diseases. No one who reads the works of Dr. Fordyce, written at the close of a long and diligent practice, can suppose that he saw diseases in the same form as at present. We find him fearful of bleeding, and prescribing bark in rheumatism and in every form of erysipelas. This may account for the few instances we meet with among the writers immediately preceding us, of a disease so highly inflammatory, and so suddenly dangerous or fatal as this kind of sore throat; and indeed for the frequency and rapid progress of many other inflammatory diseases. It should, however, in our opinion, lessen our respect for nosology; lest it should lead the young practitioner to prescribe for names and not for symptoms.

*Account of a Case of Croup, in which the Operation of Bronchotomy was successfully performed.* By THOMAS CHEVALIER, Esq. &c.

If inflammation of the larynx has been less noticed, we are obliged to the northern physicians for first pointing out to us a disease in the trachea, which is now so common that we cannot fail to express our surprize it should have been so long unnoticed. Probably, however, like the subject of the former article, it may have encreased from the same causes as have encreased the number and violence of other inflammatory diseases. As the disease seldom fails to affect the larynx with the trachea, it may, perhaps, hereafter be found that it no way differs from cynanche laryngea, excepting in the age of the subject; the effusion of lymph, or adhesive inflammation, being more common in the earlier periods of life. We offer this only as a suggestion, and hasten to give due credit to the courage and skill of the operator on so delicate a part. It is the more necessary that this case should be recorded, because it shows that after the coagulated lymph or membranous substance, which characterizes the disease, is rejected, the patient is by no means secure. The complaint is only changed to bronchitis; and the danger of suffocation from another cause remains, especially in children, who are less ready than grown people at assisting themselves by expectoration. This patient was seven years old.

"On the afternoon of Wednesday the 27th, (the day after the membranous substance was thrown up,) the difficulty of breathing greatly increased, the countenance grew livid, cold sweats came on, and he appeared sinking. He was, neverthelss, perfectly sensible. No chance of his recovery now seemed to remain, unless it were by opening the trachea, and I was applied to for this purpose. I exposed the trachea just below the cricoid cartilage, and divided two of the cartilaginous rings vertically, cutting afterwards transversely in the interstice between them. About an ounce, or an ounce and a half, of a reddish brown and frothy mucus gushed out through the opening; and, by a tolerably full inspiration, which presently followed, the child was enabled to cough up more of the same kind. The breathing became immediately relieved, the cold sweat ceased, and the countenance in a short time resumed much of its natural appearance. The pulse was 160. On the morning of the 28th the breathing was much improved: pulse 144. He was directed to take half a drachm of oxymel of squill every hour in a little camphor mixture; and four ounces of the camphor mixture were to be employed as an enema, several times in the course of the day. In the evening his breathing had become quite easy, and the cough much less sonorous. On the 29th he brought up with a slight cough, about a dessert spoonful of tough mucus, but it did not appear that any had passed through the wound. He was better in all respects, and from this time his recovery was gradual and uniform."

---

*Practical Observations on the Cure of Wounds and Ulcers on the Legs, without Rest; illustrated with Cases.* By Thomas Whately, Member of the Royal College of Surgeons in London. 2d edit. 8vo. Callow. London, 1816.

Though we have not found any considerable additions in this new impression, yet, as the first was only slightly noticed, the importance of the subject, and the respectability of Mr. Whately's name, induce us to enter more minutely into that gentleman's claims to the improvement in this branch of surgery. We are the more anxious to do this, because, highly as we have estimated his merits, it is only in his Treatise on the Affection of the Tibia, induced by Fever, that we have given him full credit for a considerable share of invention.

The preface commences with remarking, that Wiseman was well acquainted with the advantage of pressure in ulcers of the lower extremities, "The use of the laced stocking (says Mr. W.) was recommended by him for this purpose; nor can there be any doubt, that the good effects of it in his hands were very manifest. His ideas, however, seem not to have been much regarded by succeeding surgeons. We

4 find find but little said by the writers on surgery, on the effects of pressure in the cure of ulcers on the lower extremities, previous to the appearance of Dr. Underwood's treatise. Yet I am aware, that there always have been practitioners who were acquainted with the importance of this mode of treatment, and have adopted it in their practice. I had myself an opportunity of seeing the extraordinary success attending it, during my apprenticeship in the country. It is matter of fact, however, that the practice is very far from being general. Even in one of the latest publications on the subject, and this too by a surgeon of the first eminence, the effect of pressure is not much relied upon for the cure of these complaints. It is indeed there stated in several passages, not only that no benefit is derived from compression in several species of these ulcers, but that many ulcers are rendered worse, more painful, and more unhealthy in their appearance, by its use.* That there are certain conditions of an ulcer, which will not bear compression, I have allowed, and have endeavoured to point out the proper treatment, to bring on a fit state for the application of that pressure; but that an experienced surgeon should pass over so slightly this most essential part of the cure, and even speak of it as *frequently* injurious, is a circumstance hardly to be attributed to any other cause, than that of a careless and ineffectual application of the bandages. For my own part, having now been for twenty years constantly in the habit of treating a very large number of these cases, I can speak so confidently of the good effects of pressure, properly applied, that I can venture to affirm, that he who doubts of its efficacy has never given it a fair trial.

"In the cases which are added to this essay, very little variety of dressing was used; the cure was almost always trusted principally to the pressure made on the limb, under the exceptions particularly specified in the work. My success has been so uniform, that I cannot but be anxious to see this practice become established, and generally followed. Nothing but a conviction that in promoting this end, I am really doing an important service to my fellow-creatures, could have induced me to appear before the tribunal of the public, conscious as I am of my incompetency as a writer. But may I not hope, that the plain tale of a practical man will be heard, though not told with the graces of elegant language.

"In whatever manner this attempt be received, I cannot

"* See Home on Ulcers of the Legs."

 doubt doubt that the practice here recommended must, in the end, prevail, notwithstanding it has this great obstacle to contend with, that surgeons must condescend, for the most part, to apply the bandages with their own hands. The clumsy and ineffectual manner in which this business is too frequently done, can never be expected to produce the desired effect. I am certain that if the necessary pains be taken, according to the directions here laid down, such effects will uniformly follow, as must convince the unprejudiced mind, that to have recourse to the operation of tying varicose veins, and the application of a great variety of remedies, can be *very rarely*, most probably *never* necessary. I can safely declare, that all such cases as are described by Mr. Home* to be cured by this operation, have readily yielded under the proper management of pressure alone."

The author next adverts to Mr. Baynton's new method of treating with plaister bandages, which, it appears, was published whilst the first edition was in the press. After paying every proper compliment to that gentleman, Mr. W. concludes by a preference of his own plan, on account of the facility with which rollers are applied, their cheapness, and greater cleanliness.

It must be admitted, that, judging by the general, we may add almost permanent, success of the various plans, if Sir Everard Home's has fallen into entire disuse, Mr. Baynton's has retained its ground for a length of time, and with an increasing celebrity, which seems to insure its success. Mr. W. very feelingly regrets the tardy progress of both, particularly of his own, when he complains that the custom of confining patients with sore legs to an horizontal posture is still prevalent in hospitals, where their room is so much more wanted for other much worse cases; and that, where pressure has been adopted, it has been principally on Mr. Baynton's plan. This preface concludes with a paragraph which cannot be too generally attended to.

" We are told, that, in Paris, no student is admitted to the office of a dresser at any hospital, without having first given proof, in an examination, of his possessing industry and abilities sufficient to qualify him for so important an office. It would be well if such a regulation were enforced in London, where a dresser requires no other qualification than the ability to pay a certain sum of money. The general adoption of the method of cure recommended in this treatise, with the assistance of qualified dressers, would afford a great relief to the hospitals, by dressing as out-patients numbers

"* Ibid."

who who are now confined to the house, and occupy that room which is wanted for more dangerous cases. By these means students would become more adroit in this important branch of surgery; and the knowledge of the most successful management of these troublesome cases would be more generally diffused, to the credit of the profession, and the unspeakable advantage of the suffering patients."

To these remarks we shall take the liberty of adding one more. The additional fee expected by the hospital surgeon for a dressing pupil very much confines that department to young men of some independent property or expectations. These have also London connections, and superfluous cash, either of which is a sufficient inducement to engage in the dissipation of this great town. Hence their hospital duty is either neglected, or transferred to a more prudent or poorer student. Though the patients may not seem to suffer by such a change, yet many inconveniencies arise from the unsettled state of the business. When the surgeon goes round, it is in vain for him to make any inquiries of the person to whom he committed the charge of his patients, and who, unwilling to acknowledge his own inattention, can neither ask the opinion of his principal, nor satisfy him concerning the probable causes of those changes which he perceives in some of the most interesting cases.

In this preface to the second edition, the author remarks that he has not added to the number of well-authenticated cases, because he conceived the former were sufficient; otherwise he might have swelled his book by thousands of fresh histories. Of this we have no reason to doubt; and few of our readers will require more than 150 cases for the confirmation of a practice much more novel than the present. At the end of these is an appendix, in answer to some remarks contained in a second edition of Mr. Baynton's work. We shall not enter on this controversy, from the respect we bear to both the gentlemen, and because we wish our readers to try both plans, before they make up their minds; or perhaps the different states of ulcers, the difference of constitutions and of habits of life, and the season of the year, may induce a preference for one or the other, according to the above-mentioned circumstances.

A postscript follows, which we shall transcribe—

"In reprinting the preceding essay, the author forgot to insert, in its proper place, a note respecting a slight alteration in the treatment of some wounds and ulcers on the legs. In different parts of this work it is observed, that when these complaints are attended with much inflammation and pain, it is extremely improper to advise the use of pressure, or to exercise the limb; and that that rest, the horizontal position, and emollient poultices,* during this period of the disease, become absolutely necessary. I intended to have added, that when the condition of the wound or ulcer has been improved by this treatment, though not sufficiently so to render it as yet safe to apply the full pressure of the compresses and roller as before directed, or to permit the limb to be freely used, experience, in a very large number of cases, has convinced me, that a moderate degree of exercise may be allowed, not only without detriment, but sometimes with manifest advantage, provided the limb be properly supported by the roller only, without compresses, applied over the emollient poultice and mild dressing, so as to make a gentle and equable pressure upon the whole limb, from the toes to the knee, making the poultice, by this treatment, to act as a gentle compress. Most persons afflicted with these complaints, whatever may be their station in life, are extremely desirous of being relieved, as soon as possible, from a strict confinement; and such I have, for many years past, been in the habit of indulging with the moderate use of their limbs, when supported in this gentle manner, till the parts will bear full compression. It is absolutely necessary, in a great number of irritable sores, attended with inflammation, to persevere in this plan of treatment for a considerable length of time; and even after the inflammation is so far subdued that the roller can be advantageously applied with compresses through the day, it will be useful, for a short time, to have recourse to poultices during the night.

"By a careful attention to these and other rules laid down in the preceding essay, many of the prejudices against compression will be obviated. I have never seen a case where it may not be used, if well timed, to the greatest advantage."

Such are the principal additions to this new impression: they are not sufficient to render it necessary for those who are in possession of the former; but we need not urge the utility of the entire work to any of our readers.

---

### MEDICAL AND PHILOSOPHICAL INTELLIGENCE.

*ROYAL SOCIETY.*—On Thursday, the 21st of March, a paper by SIR EVERARD HOME was read, on the mode of action of specific medicines. From experiments already made it is known that poisonous bodies, whether mineral or vegetable, do not produce their effects upon the body till they are introduced

---

* Mr. W. prefers the common poultice of bread, milk, and lard, or salad oil, to the linseed meal. His reasons are very just, but, on account of the difficulty of making the domestic poultice of a sufficient consistency, we conceive a small addition of dry linseed meal is extremely useful.—EDIT.

into

